# Nanosecond laser induces proliferation and improved cellular health within the retinal pigment epithelium

**DOI:** 10.3389/fmed.2025.1516900

**Published:** 2025-03-03

**Authors:** Andrew I. Jobling, Quan Findlay, Ursula Greferath, Kirstan A. Vessey, Satya Gunnam, Victoria Morrison, Gene Venables, Robyn H. Guymer, Erica L. Fletcher

**Affiliations:** ^1^Department of Anatomy and Physiology, The University of Melbourne, Parkville, VIC, Australia; ^2^Centre for Eye Research Australia, Royal Victorian Eye and Ear Hospital, East Melbourne, VIC, Australia; ^3^Ophthalmology, Department of Surgery, The University of Melbourne, Parkville, VIC, Australia

**Keywords:** age related macular degeneration, nanosecond laser, retinal pigment epithelium, proliferation, aging

## Abstract

**Background:**

Age-related macular degeneration (AMD) is a leading cause of vision loss in those over 60 years of age. Although there are limited interventions that may prevent the development or progression of disease, more efficacious treatments are required. Short-pulsed laser treatment shows promise in delaying progression of early disease. This work details how nanosecond laser influences the retinal pigment epithelium (RPE), the principal cell type implicated in AMD.

**Methods:**

C57BL/6J mice (3-month-old) underwent monocular nanosecond laser treatment to assess short-term RPE response, while 9-month-old C57BL/6J and ApoEnull mice were similarly treated and longer-term responses investigated after 3 months. Human tissue was also obtained after 2 nanosecond laser treatments (1 month apart). RPE proliferation was assessed using bromodeoxyuridine and RPE gene change explored using qPCR and RNAseq. Melanin and lipofuscin content were quantified using histological techniques.

**Results:**

Nanosecond laser induced RPE proliferation in treated and fellow mouse eyes, with monolayer repair occurring within 3 days. This was replicated in human tissue, albeit over a longer duration (1–4 weeks). Wildtype animals showed no overt change in RPE gene expression after short or longer post-treatment durations, while laser treated ApoEnull animals showed increased *Mertk* and *Pedf* expression, and a reduced number of dysregulated aging genes in treated and fellow eyes after 3 months. Furthermore, melanin and lipofuscin content were restored to wildtype levels in laser-treated ApoEnull RPE, while melanolipofuscin granules were reduced within treated regions of human RPE.

**Conclusion:**

This work shows nanosecond laser stimulates RPE proliferation and results in an improved cellular phenotype. These data provide a biological basis for the prophylactic use of nanosecond lasers in AMD.

## 1 Introduction

Age related macular degeneration (AMD) is a leading cause of irreversible vision loss in those over 60 years of age in developed nations (1). Although there are treatments that reduce vision loss in those with advanced AMD, with the exception of dietary advice and vitamin and mineral supplementation, there are currently no therapies that reduce the progression of AMD from its earliest forms. The retinal pigment epithelium (RPE) is a single layer of post-mitotic cells situated in the posterior eye, between the choroid and photoreceptors, that plays a critical role in maintenance of retinal function (2). Specifically, the RPE controls the ionic environment within the subretinal space, is critical for recycling and phagocytosis of shed photoreceptor outer segments, absorbs stray light, and controls waste removal from the outer retina (3–5). Importantly, age-dependent and pathological changes within the RPE are thought to contribute to the development of AMD (6, 7). Therefore, therapies that aim to protect, revitalize or replenish the RPE may hold potential for preventing and/or slowing the development or progression of AMD.

The use of ophthalmic lasers as a potential treatment for AMD has been advocated since an initial study by Gass showed resolution of drusen, the hallmark feature of early AMD (8). However, the earliest studies used continuous wave lasers that caused thermally-induced retinal damage in addition to RPE ablation and in some trials was thought to be associated with AMD progression (9). Consequently, laser therapy as a means of reducing AMD progression was largely abandoned. More recently, subthreshold short pulsed laser treatments that specifically target the RPE and do not cause thermally induced collateral tissue damage have been explored (10–12). These short pulsed treatments such as the nanosecond laser achieve their RPE selectivity by delivering laser energy in short nanosecond pulses that are thought to generate microbubbles which ultimately result in membrane rupture within defined RPE cells (13). The use of subthreshold nanosecond laser treatment has been the subject of a multicenter trial (the Laser Intervention in the Early Stages of AMD, LEAD) involving 292 individuals with bilateral drusen (<125 μm). While the study found no significant effect of treatment on the progression to the development of late AMD, a subset of participants (those without reticular pseudodrusen) showed a nearly 4-fold reduction in the rate of progression to late AMD (14, 15). Importantly, these results highlight that more work is needed to fully understand how the nanosecond laser influences the posterior eye so that therapy can be appropriately targeted.

While the clinical benefit of subthreshold short pulsed laser therapy for treatment of AMD remains to be fully validated, work in animal models has provided some evidence that this type of treatment impacts some features of AMD pathology. Bruch’s membrane, a pentameric membrane that separates the RPE from its underlying vasculature, thickens during AMD development and is thought to impact nutrient/waste transfer (1). Using mouse models of AMD that have a thickened Bruch’s membrane (ApoEnull and Nrf2null), our work and that of others show that short pulsed laser treatment lead to thinning of Bruch’s membrane after 1 and 3 months post-treatment (11, 16, 17). In the case of the nanosecond laser treatment, this thinning was associated with increased *Mmp2* and *Mmp3* expression within the RPE/choroid (11). However, the mechanism(s) by which nanosecond laser impacts RPE integrity and other biomarkers of AMD development are not well known.

One possible explanation for the improved health of the posterior eye following nanosecond laser treatment may be the induction of RPE cell proliferation. RPE cells are terminally differentiated cells and exhibit age-related accumulation of the waste product, lipofuscin with time. Our previous work showed that in response to nanosecond laser treatment, some RPE cells label for the proliferation marker, Cyclin D1, implying that creation of daughter cells could potentially lead to improved RPE function following laser treatment. However, detailed evidence for RPE proliferation and the impact this has on RPE function and integrity, especially age associated accumulation of waste products, is poorly understood.

The central aim of this study was to evaluate how nanosecond laser alters RPE structure and function in a period immediately after laser application and also following a longer period. Specifically, we quantified in detail the RPE proliferative response following nanosecond laser treatment of wild type mice. This response was also validated in a human eye that had previously received 2 nanosecond laser treatments 1 month apart. We then evaluated the impact laser induced RPE proliferation has on RPE integrity by evaluating the expression of key genes. We examined longer-term changes in RPE gene expression and cellular health using RNAseq and quantitative PCR in aged wildtype and ApoEnull animals. Finally, melanin granules and lipofuscin content were quantified in laser-treated mouse and human RPE tissue.

## 2 Methods

### 2.1 Animal studies

Adult C57BL/6J mice (Animal Resources Centre, Perth, Australia) were used at 3 months of age to assess short term RPE response following nanosecond laser treatment, while 9-month-old C57BL/6J and ApoEnull (Animal Resources Centre) mice were also treated with nanosecond laser and then aged to 12 months of age to assess longer term benefits on RPE health (18). Prior to laser treatment, all animals were anaesthetized (ketamine:xylazine 67:13 mg/kg) and had topical corneal anesthesia (0.5% Alcaine; Alcon Laboratories, Switzerland) and dilation (1% atropine sulfate; Alcon Laboratories) applied bilaterally. Each mouse, regardless of age or genotype, received 10 nanosecond laser spots (each 0.065 mJ, 3 ns duration at 532 nm, 2RT^®^ laser; Ellex, Adelaide, Australia) applied unilaterally in a concentric pattern around the optic nerve head. Similar to our previous work in humans and rodents, the laser energy was determined as the dose below that observed to elicit a retinal response (11, 14, 15, 19). Specific laser treatment sites were confined to the central retina due to the optics of the mouse eye and delivered in areas that lacked retinal arterioles. The untreated fellow eye served as a within animal control, while non-lasered (naïve) controls were also included in all studies. All animals were housed in a temperature- and humidity-controlled room, with an alternating 12-h light/dark cycle with access to food and water *ad libitum*. This study was approved by the University of Melbourne Animal Ethics Committee (#1614030), and all animals were treated in accordance with the ARVO Statement for the Use of Animals in Ophthalmic and Vision Research.

The immediate, short term effects of nanosecond laser treatment on the RPE were evaluated one to 14 days after laser treatment in C57BL/6J mice. The robustness of the laser application was confirmed while animals were anaesthetized (ketamine:xylazine 67:13 mg/kg) by evaluating the retinal fundus using a Micron III fundus camera and Optical Coherence Tomography (Phoenix-Micron OR, United States). While under anaesethetic, animals were euthanized by cervical dislocation. The posterior eye cups were collected at 1-, 3-, 7-, and 14-days post-laser and either placed in a fixative containing 4% paraformaldehyde in 0.1 M phosphate buffer (PB, pH 7.4) or placed in a lysis buffer (RLT, Qiagen, Hilden, Germany) and stored at −80°C until use. In order to evaluate proliferation of RPE cells following laser treatment, some animals received injections of the proliferation marker, Bromodeoxyuridine (BrdU) as described below. In order to examine the longer term effects of a single application of nanosecond laser treatment on the RPE, C57BL/6J and ApoEnull were treated at 9 months of age and their tissues collected for analysis 3 months later at 12 months.

### 2.2 Human studies

Human tissue was obtained with informed consent from an 86-yr-old female whose right eye was removed as part of an exenteration for an aggressive lid malignancy (sclerosing basal cell carcinoma) (20). Prior to removal, both eyes exhibited scattered intermediate drusen (63–125 μm) in the mid periphery. The patient was treated with a nanosecond laser (2RT^®^ laser; Ellex) at 2 time points, 1 month and 1 week prior to exenteration of the eye (12 spots positioned in the posterior pole, with 6 spots positioned adjacent the superior and 6 spots adjacent the inferior vascular arcades; 532 nm, 3 ns, 0.3 mJ). After exenteration, the posterior eye cup was placed in 4% paraformaldehyde in PB for 4 h and then dissected into small pieces ~4mm^2^, processed through graded sucroses (10, 20, 30% w/v) and stored at −80°C until use (20). Approval for this study was obtained from the Human Ethics Committee of the Royal Victorian Eye and Ear Hospital (Melbourne, Australia; project #08/853H/18) and University of Melbourne (HREC# 22293) and studies were conducted in accordance with the tenets of the Declaration of Helsinki.

### 2.3 Immunocytochemistry

In order to evaluate the effects of the nanosecond laser on RPE integrity, mouse and human RPE wholemounts were processed for indirect immunocytochemistry. For RPE whole mounts, mouse and human samples were thawed and the neural retina removed. Eye cups containing RPE were washed in PB and incubated for 2 days at 4°C in respective primary antibodies (Table 1). Samples were then washed 3 times in PB, incubated in secondary antibody (Table 1) overnight at room temperature, washed in PB and finally mounted (Dako, North Sydney, Australia) for imaging. Samples were imaged using a confocal laser scanning microscope (LSM 880, Zen software; Zeiss, Jena, Germany) with 10x/0.45, 20x/0.8 objectives at a resolution of 1,024 × 1,024 pixels. Tile scans of the entire RPE surface were taken, as were limited z-stacks (6–9 μm).

**Table 1:** Antibodies used for immunocytochemistry.

Antibodies	Target	Dilution/species	Manufacturer/#cat
BrdU	Thymidine analog that labels proliferating cells	1:100, rat	Abcam, # 6326
Ki67	A nuclear protein that labels proliferating cells	1:200, rat	Invitrogen, # 14-5698-82
Cyclin D1	Cell cycle regulatory protein that labels proliferating cells	1:5, rabbit	Abcam, # Ab21699
Alexa Fluor 568-Peanut agglutinin	A lectin that labels cone photoreceptors	1:100	Life Technologies, #L32458
Calbindin	Calcium-binding protein that labels horizontal cells	1:4000, mouse	Swant, #AB300
Protein Kinase Cα	A serine/threonine kinase that labels rod bipolar cells	1:500, mouse	Sigma, P5704
Alexa Fluor 633-Phalloidin	Polymerized actin filaments, that labels RPE cell boundaries	1:200	Life Technologies, # A22284
Bisbenzimide	Nuclei stain	1:1000	Sigma-Aldrich, # 14530
4′,6-Diamidino-2-Phenylindole, Dihydrochloride (DAPI)	Nuclei stain	1:1000	Invitrogen, #D1306
Donkey anti-rat Alexa 488	Secondary antibody	1:100	Life Technologies, # A21208
Goat anti-mouse IgG Alexa 488	Secondary antibody	1:500	Life Technologies, # A21206
Goat anti-rabbit IgG Alexa 594	Secondary antibody	1:500	Life Technologies, # 21207

The above antibodies were used to explore the effects of nanosecond laser treatment on mouse and human eyes. The targets, dilutions and provider details for the various antibodies are listed.

For vertical sections, eye cups were thawed and embedded in Optimal Cutting Temperature compound (Tissue-Teka; Sakura, Torrance, CA), frozen at −20°C and sectioned transversely at 12 μm on a cryostat (Microm HM550, Thermo Scientific, Walldorf, Germany). Sections were placed onto polylysine-coated slides (Thermo Scientific, Melbourne, Australia) and stored at −20°C. Frozen sections were washed in PB and incubated with primary antibody (Table 1) overnight at room temperature. Following washing in PB, samples were incubated with secondary antibodies (Table 1) for 1 h, washed and mounted as above. The nuclei stain, Bisbenzimide (1:1000, Sigma-Aldrich), was included and images were acquired as above.

### 2.4 RPE cell proliferation

In order to examine whether nanosecond laser treatment induced proliferation of RPE cells, labeling of RPE cells following treatment with the S-phase marker, following treatment with the S-phase marker, BrdU was quantified. Animals received BrdU (100 mg/kg, intraperitoneal injection) twice a day (10 am and 3 pm) for different durations after laser treatment to investigate the temporal proliferation rate. Animals received BrdU for either 1 day (day 0–1 post-laser, *n* = 6), 2 days (day 1–3 post-laser, *n* = 6), 4 days (day 3–7 post-laser, *n* = 6) and 7 days (day 7–14 post-laser, *n* = 6), with treated and fellow posterior eyecups collected at 1-, 3-, 7-, and 14-days post-treatment. Naïve control animals that did not undergo nanosecond laser treatment received BrdU injections for 4 and 7 days (days 3–7 and 7–14 post-laser, respectively) and posterior eyecups were collected.

Mouse tissues were fixed in 4% paraformaldehyde in PB for 30 min, washed x3 in PB and cryoprotected (10, 20, and 30% sucrose in PB). Tissues remained in 30% sucrose overnight at 4°C before being stored at −30°C until use. Prior to BrdU immunolabeling, mouse posterior eyecups were exposed to DNase (150 U/mL; Sigma Aldrich) for 2 h at 37°C, inactivated in 50 mM Tris–HCL (pH 7.6) at 70°C for 10 min, transferred into chilled Tris–HCL for 3 min and finally washed in PB. The eyecups were subsequently exposed to 2 N HCL for 45 min at 37°C and then washed three times with PB, before being neutralized by 0.2 M sodium tetraborate for 10 min. Tissues were finally washed x3 in PB, before being processed for BrdU immunocytochemistry as described below.

### 2.5 Quantification of RPE number and proliferation following nanosecond laser

RPE cell density within laser treated regions was quantified by counting the number of cells in a 200 μm diameter circle centered on each laser lesion. A circle of 200 μm diameter was chosen for quantification because nanosecond laser treatment is known to induce a circular lesion of this diameter. All cells within each 200 μm diameter circle were quantified. Those cells that intersected the circle were also counted if more than 50% of their cell body fell within circle. For quantification of RPE proliferation, the number of BrdU-labeled cells were counted within the laser-treated regions. The proliferation of RPE cells in non-laser treated regions of the treated eye were quantified by counting BrdU-labeled nuclei across the whole eye cup, excluding those present within the laser-treated region. The proliferation of RPE cells in the fellow eye and in control eyes was quantified by counting BrdU-labeled cells across the entire posterior eyecup. RPE proliferation rate was calculated as the number of BrdU cells/mm^2^ RPE per day.

A second marker, Ki67, was used to confirm RPE proliferation. Immunolabeling for Ki67 was quantified 3 days following nanosecond laser treatment and the number of Ki67 immunoreactive cells /mm^2^ RPE quantified.

### 2.6 RNA sequencing and quantitative gene expression

Quantitative PCR analysis was performed on short term (C57BL/6J, 1-, 3-, 7-days post laser) and aged (C57BL/6J and ApoEnull, 12 month) samples. Total RNA was isolated from RPE/choroidal samples (*n* = 9 per group; RNeasy micro, Qiagen, reverse transcribed (Sensiscript RT, Qiagen) and amplified (Sensifast SYBR, Meridian Bioscience, Cincinnati, OH) using the primers shown in Table 2. Absolute gene expression was quantified with reference to external standards for each gene of interest and the respective housekeeping genes, hypoxanthine phosphoribosyltransferase, *Hprt* and glyceraldehyde-3-phosphate dehydrogenase, *Gapdh* as previously published (21). Data were presented as copies gene of interest per copy of *Hprt*. For qPCR array analysis, 3 aged RPE/choroidal samples were pooled per experiment and 3 independent experiments performed (*n* = 3/group). RNA samples were pre-amplified, reverse transcribed and the expression of 84 aging-related genes quantified using a commercial array kit (RT^2^ Profiler PCR Aging array, Qiagen) and data expressed relative to the C57BL/6J fellow eye using 2^−ΔΔct^ (22).

**Table 2:** Oligonucleotide primer sequences.

Gene	Sequence	Forward primer	Reverse primer	Product size (bp)
*Rpe65*	NM_029987.2	GTTGCTGGAAAGGGTTTGAA	CAGTTGTATGGGGCAGTGTG	186
*Mertk*	NM_008587.2	CGGGGCTAGACATGAACATT	GTGTGACTGCAGCAAAAGGA	180
*Pedf*	NM_011340.3	TCATTCACCGGGCTCTCTAC	GCCTGCACCCAGTTGTTAAT	250
*Bdnf*	NM_007540.4	GCGGCAGATAAAAAGACTGC	CTTATGAATCGCCAGCCAAT	248
*Hprt*	NM_013556.2	CCTAAGATGAGCGCAAGTTGAA	CCACAGGACTAGAACACCTGCTAA	86
*Gapdh*	NM_008084.2	TGTGTCCGTCGTGGATCTGA	TTGCTGTTGAAGTCGCAGGAG	150

Primers were designed relative to the respective published mouse sequences, are shown 5′ to 3′ and the product sizes are included. For the generation of external standards, the primers contained a T7 promoter sequence at the 5′ end of the forward primers, while a poly T_15_ sequence was included at the 5′ end of the reverse primers.

Aged C57BL/6J and ApoEnull mice (9 months old) were treated with nanosecond laser as mentioned above in section 2.1, allowed to recover and were assessed after 3 months. Animals were sacrificed and posterior eye cups (laser treated, fellow eye and naïve control) isolated from which the neural retina was removed. RPE/choroidal samples were isolated by the addition of a lysis buffer and total RNA isolated (RNeasy micro, Qiagen). For aged C57BL/6J samples (laser treated and naïve control groups, *n* = 6 per group) a SmartSeq v4 kit (Clontech Laboratories, Mountain View, CA) was used to pre-amplify the samples prior to library preparation and RNA-Seq (Walter and Eliza Hall Institute of Medical Research, Melbourne, Australia). Basic bioinformatic analysis (differential gene expression) was performed by the Australian Genome Research Facility (Melbourne, Australia), while gene set enrichment analysis (GSEA) and leading edge analysis were performed using the GSEA v4.3.2 (Broad Institute, Cambridge MA) according to a previously published pipeline of bioinformatic analysis (23). Enrichment was evaluated across the mouse Hallmark (containing 50 gene sets) and mouse C2-reactome (containing 4,738 gene sets) from the Molecular signature database.

### 2.7 Transmission electron microscopy and melanosome/lipofuscin quantification

Transmission scanning electron microscopy was used to quantify melanin and lipofuscin content in the RPE from nanosecond laser treated aged C57BL/6J and ApoEnull animals. Posterior eye cups were isolated and processed as previously described (11, 24). Ultrathin sections (70 nm) were contrasted with uranyl acetate and lead citrate solutions and imaged on a Phillips CM120 electron microscope (FEI, Hillsboro, OR). Melanin content and lipofuscin area were measured in RPE cells and quantified as a measure of length of field/transverse cell area, respectively. Image analysis was undertaken as per our previous work and according to the melanin/lipofuscin identification specified in Vessey et al. (24) and Julien et al. (25). At least five micrographs were analyzed per animal and the results averaged (*n* = 3 animals per genotype and treatment group).

Lipofuscin and melanolipofuscin content were quantified from human RPE cells within laser treated (post 1-month) and adjacent non-treated areas. High magnification images (63 x oil, 680 nm z thickness, 2,432 x 2,432pixel, LSM 880; Zeiss with airyscan mode) were taken of single RPE cells, which were stained with phalloidin-633 (RPE cell membrane; Table 1) and bisbenzimide (RPE nuclei, Table 1). The different autoflourescent granules representing lipofuscin; melano-lipofuscin and melanosomes were distinguished using confocal microscopy and were counted and expressed relative to RPE cell area (ImageJ, NIH, Bethesda, MD) (26). A total of 12 RPE cells within laser treated regions and 17 control RPE cells (outside the laser treated regions) were quantified.

### 2.8 Statistical analysis

All quantitative data from immunohistochemical and gene expression studies were analyzed using either one-way or two-way ANOVAs, followed by the Tukey’s *post-hoc* test or Šídák’s *post-hoc* analysis where appropriate. Significance value was set at *p* < 0.05 and all statistical tests were performed using the GraphPad Prism (Version 7.00, La Jolla, CA). Data are represented as mean ± SEM unless otherwise stated in figure legends.

## 3 Results

Currently there are limited interventions that prevent the development or progression of AMD (10–12). Here, we evaluated the effects of nanosecond laser treatment on RPE cell proliferation, RPE gene expression and pigment accumulation within 2 weeks of laser application or after a more extended post treatment duration of 3 months.

### 3.1 Short term RPE response to nanosecond laser treatment

**Figure 1:**
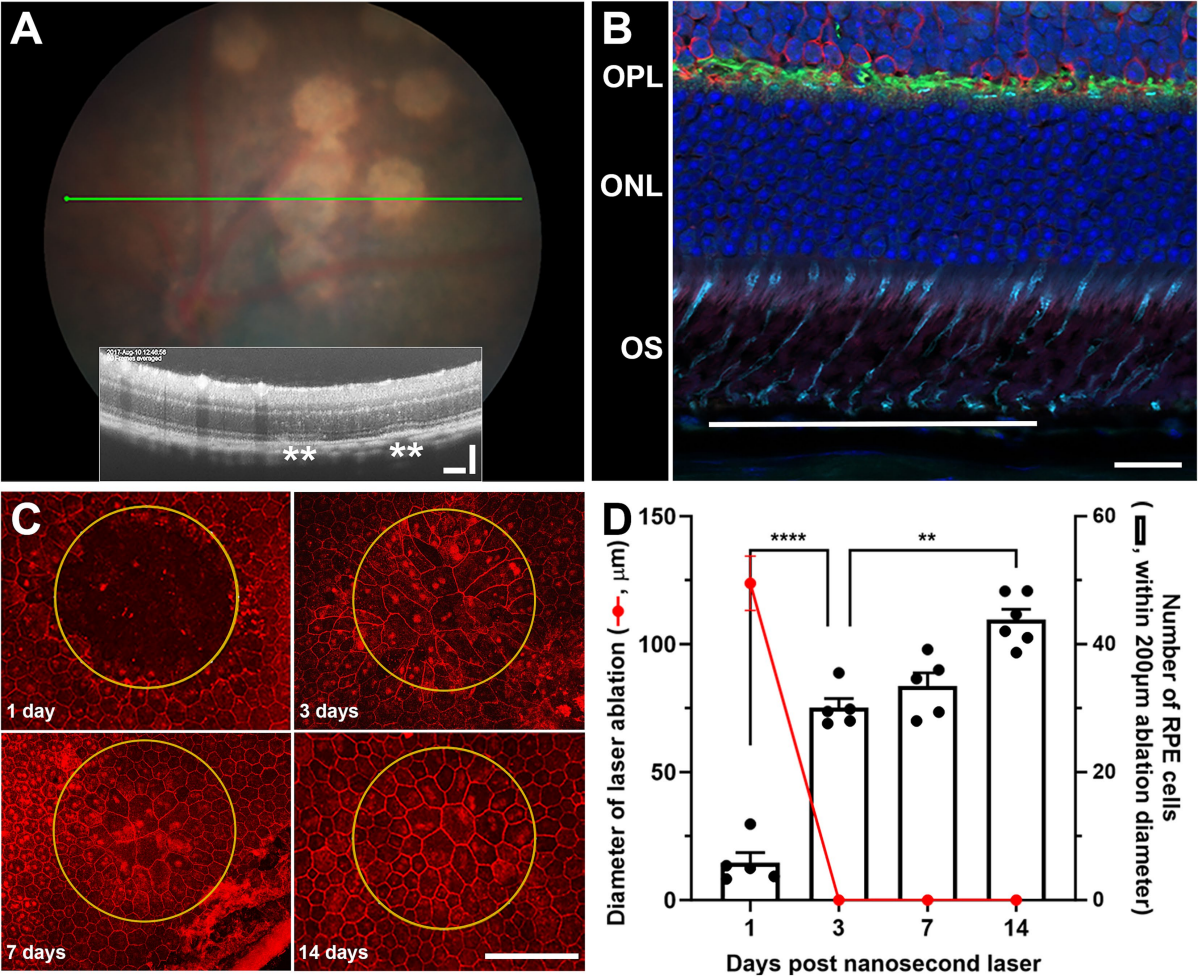
The effect of nanosecond laser treatment on retinal structure and characterization of rapid RPE healing. Wild type C57BL/6J were treated with nanosecond laser (0.065 mJ, 10 spots) and the retinal response and RPE healing assessed. **(A)** Distinct hypopigmented areas can be easily observed after nanosecond laser treatment, although OCT images through these areas (green line on fundus) show no overt retinal change and only minor disruption of the RPE layer (inset, asterisks). **(B)** Immunohistochemical assessment of retinal structure directly adjacent to the laser treated region (line) shows little alteration in cone photoreceptor (peanut agglutinin, cyan), horizontal cells (calbindin, green), rod bipolar cells (protein kinase C, red) or nuclear layers (DAPI, blue). **(C)** Staining RPE whole mounts indicates the selective ablation of the monolayer at treatment sites (yellow ring, phalloidin, red) after 1 day, while the monolayer is rapidly repaired/refined after 3, 7, and 14 days. **(D)** When quantified, the RPE monolayer is completely replaced from 3 days post-laser (red line), while the number of RPE cells within the treatment area increased significantly by 3 days and again at 7 days post-laser (one-way ANOVA, Tukey *post hoc* analysis). Data shown as mean ± SEM, *n* ≥ 5. Scale bars 20 μm **(B)**, 100 μm **(A,C)**. **p < 0.01, ****p < 0.0001. OS, outer segment; ONL, outer nuclear layer; OPL, outer plexiform layer.

Adult C57BL/6J mice were treated with a low energy nanosecond laser (2RT^©^, Ellex, 10 spots). While the energy levels used in this study were very low (0.065 mJ), and equivalent to sub-threshold levels in humans (11, 14, 15), the nanosecond laser spots can be seen in the hyperpigmented C57BL/6J fundus (Figure 1A). An OCT section through the laser treated regions (Figure 1A, inset) showed no overt alteration in retinal structure, however, a slight disruption of the RPE layer was apparent (asterisks). Supporting previous work from our group (11) and others (10), immunohistochemical staining of the retina at laser treated regions show little if any alteration in the various retinal layers, including those directly adjacent to the treated region (Figure 1B). In fact, the nanosecond laser treatment was confined to the RPE layer with discrete ablation of a 200 μm diameter region consistently observed after 1-day post-treatment (Figure 1C). The RPE monolayer was retiled by day-3 post-treatment, with evidence of ongoing RPE cell remodeling occurring over the next 11 days (day-14 post laser treatment, Figure 1C). Quantification of this healing response showed that 100% coverage was achieved after 3 days, and this correlated with an increase in RPE cell number between days 1–3 (Figure 1D; *p* < 0.0001, one-way ANOVA). A further increase in cell number was evident between 3 and 14 days post-laser (Figure 1D; *p* < 0.001, one-way ANOVA).

**Figure 2:**
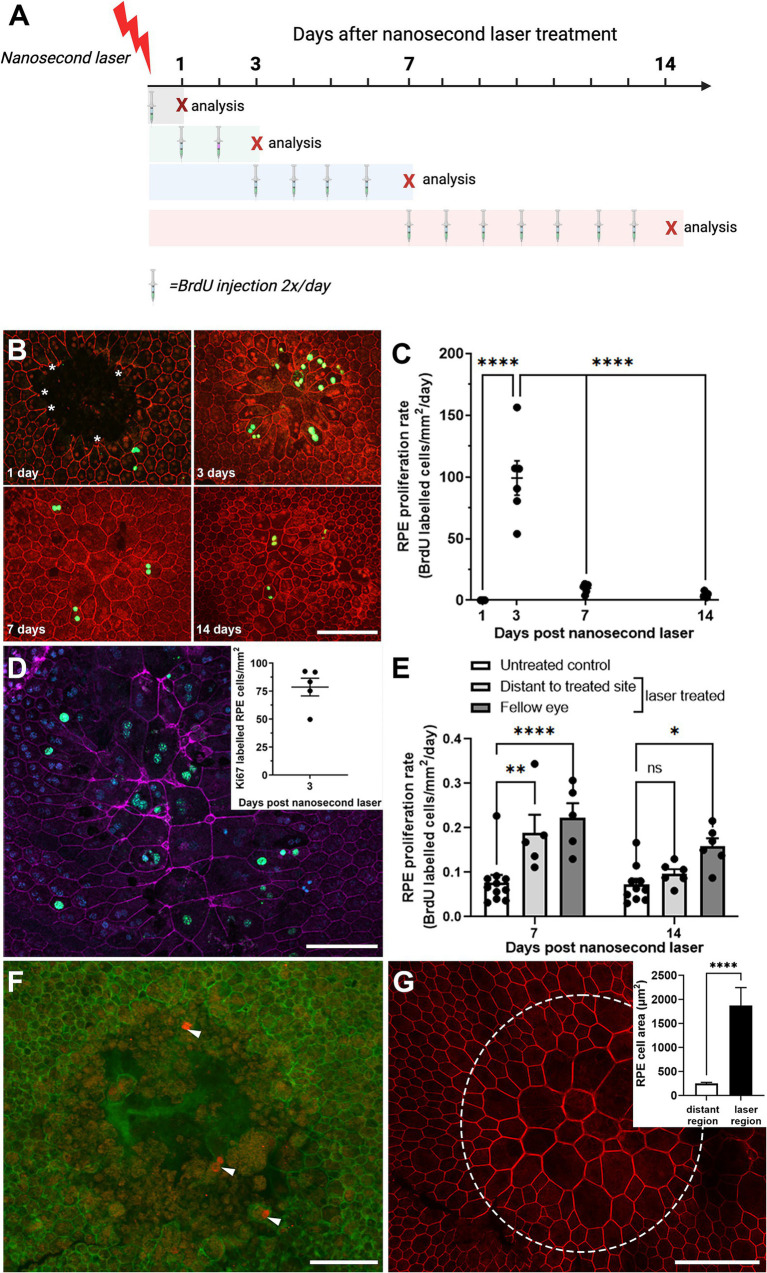
Temporal RPE cell proliferation rate after nanosecond laser treatment. **(A)** The proliferation biomarker, BrdU, was injected for various durations after nanosecond laser treatment as shown in the schematic diagram. Mice, allocated to four groups, received nanosecond laser treatment at day 0. Then mice in each of the four groups received BrdU injections immediately after laser treatment, on day 1 and 2, days 3–7 or 7–14 (injections indicated by the syringe). The “X” indicates the day after laser treatment that animals were euthanized and their tissues collected. **(B)** Immunocytochemical labeling of BrdU (green) was assessed in RPE (phalloidin, red) at various times post-laser treatment (1-, 3-, 7-, 14-days). **(C)** Graph showing proliferation rate 1, 3, 7, and 14 days after laser treatment. Proliferation rate was significantly increased at 3 days compared to all other time points (one-way ANOVA, Tukey *post hoc* analysis). **(D)** The incorporation of a second proliferation marker (Ki67, green) in RPE (phalloidin, purple; DAPI, blue) was assessed 3 days post-laser treatment with incorporation similar to that observed with BrdU (inset). **(E)** BrdU incorporation was quantified in regions distant to the laser treated site and in fellow eyes and compared to untreated controls. Increased proliferation was observed in distant regions in the treated eyes and in the untreated fellow eyes after 7 days post-laser, with this persisting in the fellow eye group after 14 days (two-way ANOVA, Tukey post hoc analysis). **(F)** When laser treatment was performed on an 86-yr-old female (1 month and again 1 week prior to exenteration), there was evidence of RPE cell proliferation (cyclin-D1, red; phalloidin, green) after 1 week of treatment. **(G)** When the earlier treatment sites were imaged (1 month post-treatment), the RPE monolayer was fully re-tiled, with larger RPE cells predominating (phallodin, red; cell area inset). Data shown as mean ± SEM, *n* ≥ 5. Scale bars 100 μm. *p < 0.05, **p < 0.01, ****p < 0.0001.

In order to evaluate the short-term proliferative response of the RPE to nanosecond laser treatment, wildtype C57BL/6J mice were subjected to nanosecond laser treatment (10 spots) and then intraperitoneally injected with BrdU for various time periods to assess the temporal proliferation rate. A summary of the experimental paradigm is shown in Figure 2A. As can be seen in the representative images, the RPE cells surrounding the ablated area (phalloidin, red) have very few BrdU labeled cells at one day post-treatment (BrdU, green), yet show evidence of select RPE cell migration (asterisks), similar to that previously described after *in vitro* nanosecond laser treatment (27). At 3 days post-laser there is an increased number of BrdU labeled cells, with reduced numbers evident at 7- and 14-days post treatment (Figure 2A). When quantified there was a significant increase in RPE proliferation rate from day 1–3 post laser treatment compared to all other times (Figure 2B, *p* < 0.0001, one-way ANOVA). In order to validate this laser-induced RPE proliferation, a second biomarker of proliferating cells, Ki67, was quantified at 3 days post-laser. Similar to the BrdU labeling, a considerable number of proliferating (Ki67 positive, green) cells were evident within the laser treated region (Figure 2C, inset shows the quantification of proliferating cells).

Interestingly, when the number of proliferating RPE cells was quantified in the laser-treated eye at regions distant from the laser spots and in the fellow untreated eye, a significant number of proliferating RPE cells were detected at both 7 post-treatment, with this extending out to 14 days post-treatment in the fellow untreated eye (Figure 2D; *p* < 0.01 time, *p* < 0.0001 eye, 2-way ANOVA). This proliferation rate was significantly lower than that observed within the laser-treated region (compare Figure 2B versus Figure 2D). Investigation of the human eye treated with nanosecond laser at two time points (1 month and 1 week prior to exenteration) was consistent with the short term RPE response in the mouse eye, showing evidence of possible RPE cell proliferation in cells bordering the treatment site at 1 week after laser treatment (Figure 2E; cyclin-D1, red). The retiling of the human RPE monolayer occurred over a longer duration compared to that observed in the mouse, with an incomplete monolayer present in 1-week post-laser lesions (Figure 2F; phalloidin, green), while the earlier treatment sites (1 month prior to exenteration) were fully re-tiled, with larger RPE cells predominating (Figure 2G and quantification shown inset).

**Figure 3:**
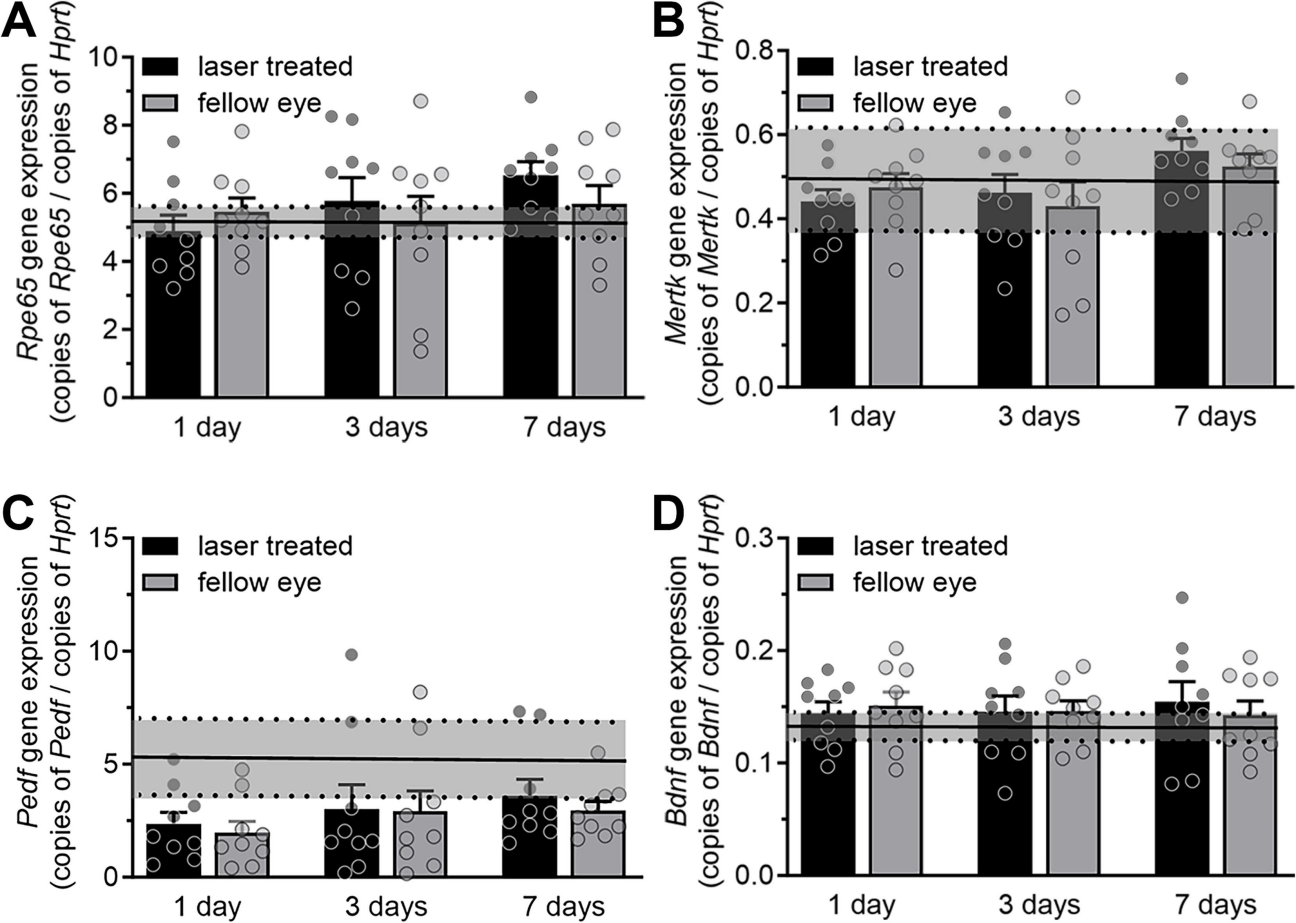
RPE gene expression after short term nanosecond laser treatment. RPE-choroid samples were isolated 1-, 3-, and 7-days post laser treatment and from a group of naïve animals. Total RNA was isolated, reverse transcribed and quantitative PCR was performed on select genes (*Rpe65*, *Mertk*, *Pedf*, *Bdnf*) and expressed relative to the housekeeping gene *Hprt*. **(A)** *Rpe65* gene expression showed no change in either the treated or fellow eyes at either 1, 3, or 7-days post laser treatment, when compared to the treatment naïve group (mean ± 95% CI, shaded region). **(B)** Similarly, *Mertk* gene expression was unaltered in treated and fellow eyes at all time points when compared to treatment naïve controls (mean ± 95% CI, shaded region). **(C)** *Pedf* expression was unaltered across all time points (compared to treatment naïve control mean ± 95% CI, shaded region, one-way ANOVA). **(D)** *Bdnf* gene expression was not altered in either treated or fellow eyes at any time point post-laser treatment (compared to naïve control mean ± 95% CI, shaded region). Data are shown as mean ± SEM, while naïve control mean (solid line) ± 95% CI (dashed lines and shaded area) are highlighted for each gene comparison. *n* = 9 per group.

As nanosecond laser treatment led to the proliferation of new RPE cells, both in the treated region and to a lesser extent in distant cells, we next investigated whether this was accompanied by an alteration in gene expression of key RPE genes involved in neuronal support. Quantitative gene expression was performed on *Rpe65*, *Mertk*, *Pedf*, and *Bdnf* in naïve, laser treated and fellow untreated RPE/choroidal samples at 1-, 3- and 7-days post nanosecond laser treatment. As is shown in Figures 3A,B, nanosecond laser did not alter *Rpe65* or *Mertk* gene expression in either the treated or fellow eyes at any time point post-laser when compared to a naïve control (*p* = 0.379 and 0.27, respectively, one-way ANOVA). Similarly, *Pedf* and *Bdnf* gene expression were unaltered at any time point post-laser treatment when compared to a naïve control (Figures 3C,D; *p* = 0.08, *p* = 0.90, respectively, one way ANOVA). Therefore, in response to nanosecond laser treatment, RPE cells rapidly proliferate to re-tile the monolayer, with the newly formed cells showing the same select gene expression profile as untreated age-matched controls. Additionally, increased RPE cell proliferation occurs in areas distant from the laser treatment site, including the fellow, untreated eye.

### 3.2 Long term response of the RPE to nanosecond laser treatment in aged C57BL/6J mice

**Figure 4:**
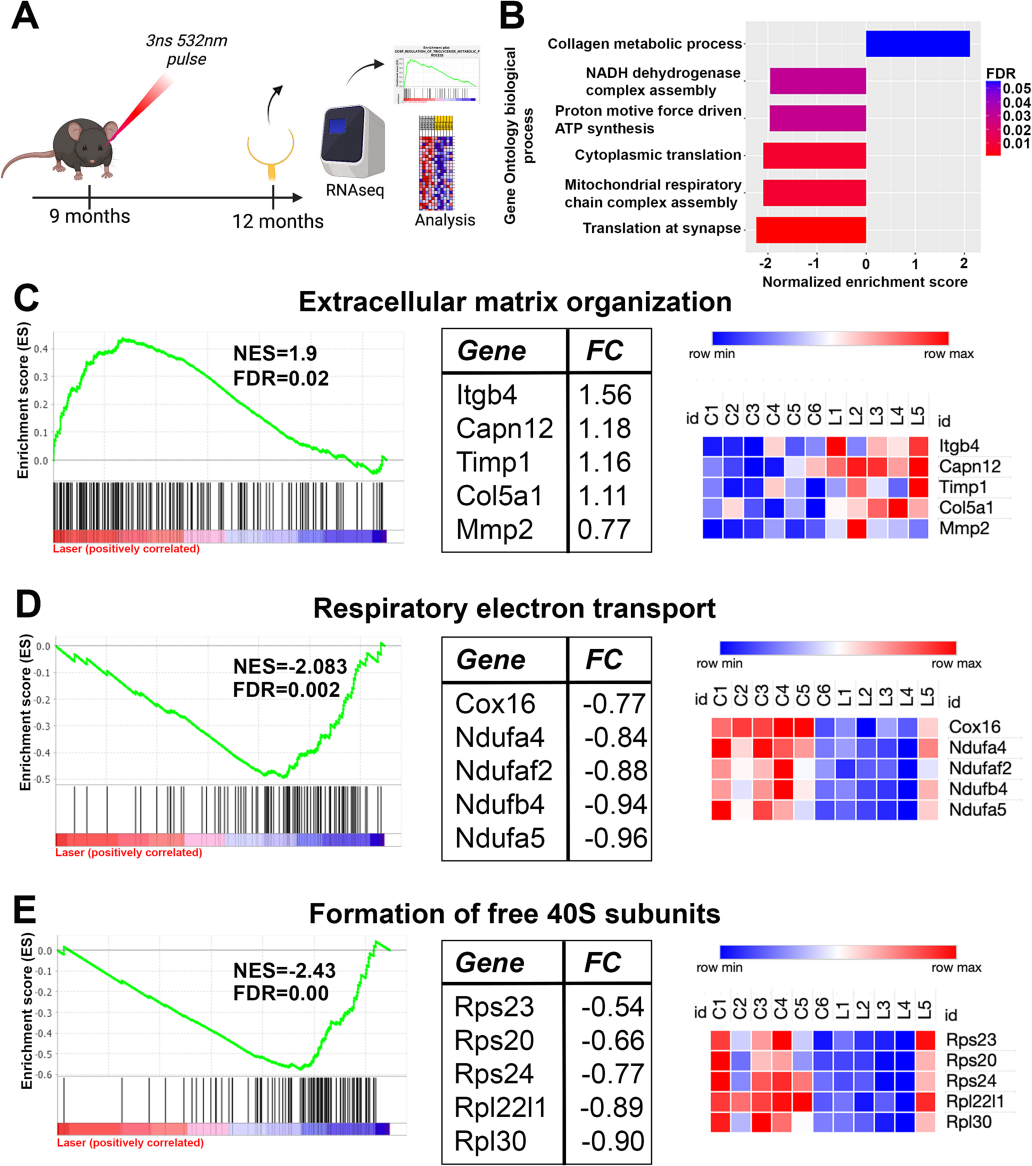
Long term gene set changes in aged RPE/choroidal transcriptome after nanosecond laser treatment. Aged C57BL/6J animals were treated with the nanosecond laser at 9 months and then RPE-choroid isolated at 12 months of age for RNAseq analysis. **(A)** Schematic diagram outlining the experimental design and timeline of procedures. **(B)** Gene ontology enrichment analysis was performed on the RNAseq dataset and the highlighted biological processes were altered by treatment with the nanosecond laser (FDR indicated by the color). **(C)** Further analysis of the dataset with curated reactome pathways showed the extracellular matrix organization reactome was significantly enriched by the laser, with the enrichment plot, top five regulated pathway genes and respective sample heatmaps shown. **(D)** The respiratory electron transport reactome (energy production) gene set was significantly negatively enriched suggesting possible altered metabolic function following laser treatment. Again, the top five regulated pathway genes and the respective samples heatmaps are included. **(E)** The formation of free 40S subunit reactome (translation) was down regulated by laser treatment as indicated by the enrichment plot, with the top five regulated genes and respective heatmaps also included. NES, normalized enrichment score; FDR, false discovery rate; FC, log_2_fold change.

The lack of a change in gene expression post nanosecond laser may reflect the fact that the above short-term study used young, wildtype animals, making it unlikely that newly produced RPE cells would show supranormal changes post-laser. As aging is known to lead to RPE cell dysfunction, and previous work by us and others suggests that treatment with nanosecond laser can produce longer term alterations in RPE gene expression and Bruch’s membrane (11, 28), we sought to explore longer term effects in older animals. Monocular nanosecond laser treatment was performed on 9-month-old C57BL/6J mice and the RPE-choroidal samples collected after 3 months for bulk RNA sequencing (see Figure 4A). We first confirmed that RPE samples collected were indeed enriched with RPE specific genes by comparing the 11 most enriched genes in our dataset with a list of RPE marker genes (Table 3) (29–31). Of the top 11 enriched genes, 10 were previously reported as RPE ‘signature genes’ in mice and humans, while the long non-coding RNA, *Malat1*, is also known to be expressed in RPE cells (32). Despite the dataset showing a high level of RPE related genes, differential gene expression showed no significant change in gene expression between the nanosecond laser treated and aged-matched naïve samples when corrected for multiple comparisons (false discovery rate, FDR > 0.05).

**Table 3:** Comparison of the top 11 genes in the RPE/choroidal RNAseq dataset with RPE signature gene sets.

Gene ID	Gene name	Average gene counts	RPE signature gene
*Rgr*	RPE-Retinal G Protein-Coupled Receptor	2,013,214	Y
*Trf*	Transferrin	1,524,172	Y
*Ptgds*	Prostaglandin D2 synthase	1,301,485	Y
*Ttr*	Transthyretin	551,278	Y
*RPE65*	Retinal pigment epithelium-specific 65 kDa protein	392,340	Y
*Clu*	Clusterin	327,035	Y
*Enpp2*	Ectonucleotide Pyrophosphatase/Phosphodiesterase 2	303,627	Y
*Malat1*	Metastasis-associated lung adenocarcinoma transcript 1	286,979	N
*Ctsd*	Cathepsin D	270,404	Y
*Arl6ip1*	ADP-ribosylation factor-like 6 interacting protein 1	262,444	Y
*Timp3*	TIMP metallopeptidase inhibitor 3	181,882	Y

The top 11 most highly genes detected in the current RNAseq study were compared to previously published RPE signature genes. While all 11 genes are known to be expressed in the RPE, 10 of 11 genes were defined as “RPE signature genes” based on previous studies (29–31).

We performed gene set enrichment analysis on the full transcriptomic dataset to examine whether specific pathways or cellular processes were altered by nanosecond laser treatment (33). Notably, gene set enrichment analysis is a method that allows the identification of collections of genes that are enriched in samples, rather than individual (significantly dysregulated) genes (33). Based on this analysis we identified several biological processes that were significantly altered by nanosecond laser (FDR < 0.05; Figure 4B). Of note, gene sets relating to extracellular matrix (collagen metabolic process) were significantly enriched, while gene sets relating to translation (cytoplasmic and synaptic) and energy pathways (mitochondrial respiratory chain assembly complex) were significantly negatively enriched. We further examined these biological processes by performing gene set enrichment using the curated reactome pathways. A total of 26 reactome pathways were significantly attenuated in laser treated RPE cells, with 9 reactome pathways relating to extracellular matrix turnover being significantly upregulated by laser treatment. The enrichment plot and normalized enrichment score (NES) is shown for the extracellular matrix organization reactome, with the top 5 genes identified in this pathway and their respective heatmaps also shown (Figure 4C). In addition, specific energy production reactome (respiratory electron transport) was negatively enriched by laser treatment, with the NES and top 5 specific genes identified (Figure 4D). Similarly, pathways relating to translation (formation of free 40S subunits) were downregulated (Figure 4E). Overall, gene enrichment analysis suggests that a single application of nanosecond laser alters select molecular pathways even after 3 months following treatment.

### 3.3 Long term response of the RPE to nanosecond laser treatment in aged ApoEnull mice

**Figure 5:**
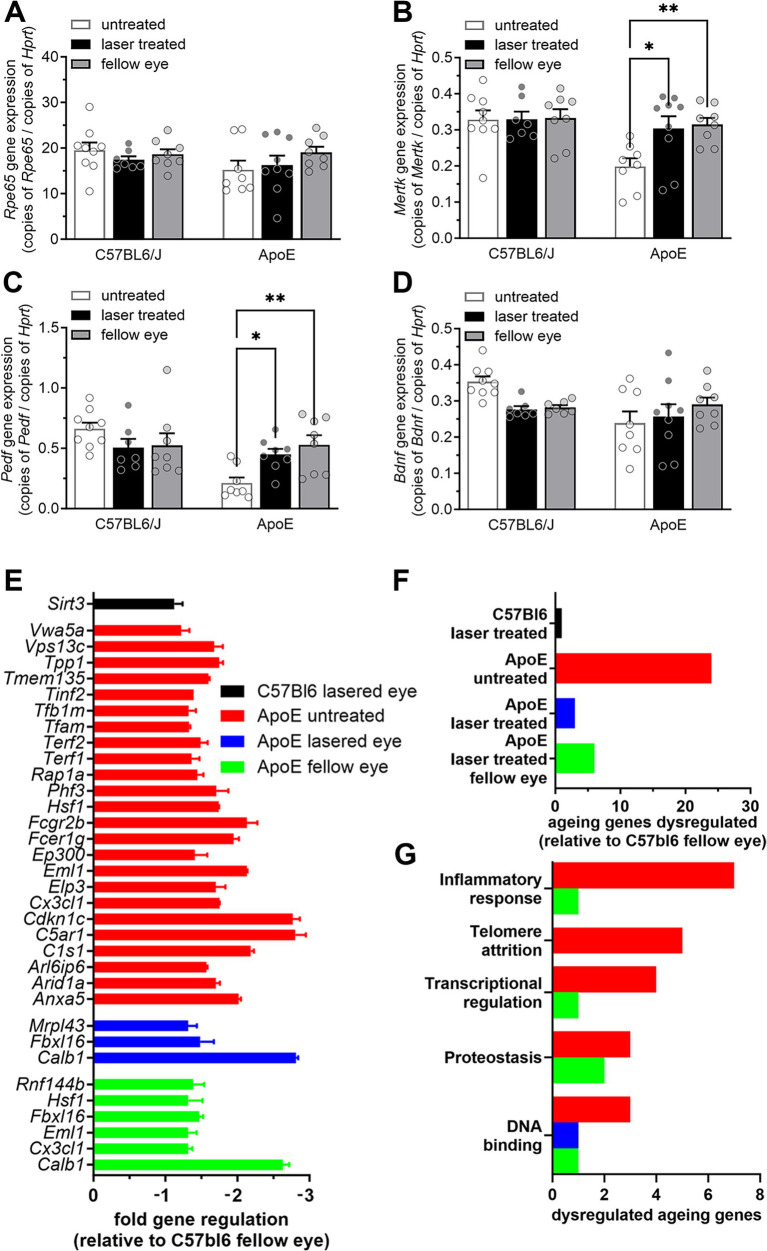
Nanosecond laser alters neuronal support gene regulation and reverses aging gene dysregulation in ApoEnull mice. Aged C57BL/6J and ApoEnull mice were treated with the nanosecond laser at 9 months of age and tissue taken at 12 months. RPE-choroid samples were isolated, total RNA isolated, reverse transcribed and quantitative PCR was performed on select genes (*Rpe65*, *Mertk*, *Pedf*, *Bdnf*) and expressed relative to the housekeeping gene *Hprt*. **(A)** *Rpe65* gene expression showed no change in any of the genotypes (C57BL/6J, ApoEnull), nor groups (naïve, laser treated, fellow eye). **(B)** While *Mertk* gene expression was unaltered any of the C57BL/6J groups, ApoEnull naïve animals showed a decrease in expression, which was restored to C57BL/6J baseline levels after laser treatment (one-way ANOVA, Tukey post hoc analysis). **(C)** A similar effect was observed for *Pedf* gene expression, with reduced ApoEnull levels increased to C57BL/6J levels in the laser treated and fellow eyes (one-way ANOVA, Tukey post hoc analysis). **(D)** *Bdnf* gene expression was not altered in either genotype, nor treatment groups. **(E)** A commercial qPCR array was performed to assess the long-term effect of nanosecond laser treatment on 84 aging-related genes. Fold gene regulation was determined relative to the C57BL/6J fellow eye and showed little change in aging genes in the laser treated C57BL/6J eyes, while numerous genes were dysregulated in the ApoEnull naïve animals (all annotated genes significant, *p* < 0.05). **(F)** Laser treatment of the ApoEnull, reduced the number of dysregulated aging genes from 24 to 3 and 6 genes in both the treated and fellow eyes, respectively. **(G)** Grouping these genes in terms of biological significance showed laser treatment to have a broad effect on genes involved in inflammation, telomere attrition and transcriptional regulation. Data are shown as mean ± SEM, *n* = 9 per group for **(A–D)**, *n* = 3 for **(E–G)**. *p < 0.05, **p < 0.01.

Having identified short term proliferative changes in the RPE and longer term effects of the nanosecond laser on select molecular signaling pathways 3 months following treatment, we next asked whether nanosecond laser resulted in long term RPE improvements in a pathological model of disease. To do this, we treated ApoEnull mice, an animal model known to develop thickening of Bruch’s membrane and altered RPE function (11, 34). C57BL/6J and ApoEnull animals aged 9 months were treated monocularly with nanosecond laser and their RPE-choroidal tissue isolated 3 months later for quantitative PCR. Similar to the short-term response, *Rpe65* and *Bdnf* gene expression were not altered by laser treatment in either the aged C57BL/6J or ApoEnull mice (Figure 5A, *p* = 0.45, Figure 5D *p* = 0.44; two-way ANOVA). By contrast, both *Mertk* and *Pedf* expression levels were reduced in the untreated ApoEnull animals, and abrogated by laser treatment (Figures 5B,C; *p* < 0.01 genotype, *p* < 0.05 laser; two-way ANOVA). This effect was observed in both the laser treated RPE and also in the untreated fellow RPE. No alterations in *Mertk* or *Pedf* expression were observed in the C57BL/6J treatment groups (*Mertk*, treated *p* = 0.999, fellow 0.99; *Pedf*, treated *p* = 0.25, fellow *p* = 0.32).

In order to gain a broader appreciation of gene expression changes in the ApoEnull RPE brought about by the nanosecond laser, a commercial qPCR array on 84 select genes known to be involved in age-related dysfunction was performed. Similar to our RNAseq dataset, nanosecond treated C57BL/6J RPE-choroidal samples showed little effect when compared to the fellow untreated eye, with only one gene significantly altered (*Sirt3*, Figures 5E,F). By contrast, the ApoEnull untreated samples showed 24 down regulated genes, including *C1s1*, *C5ar1*, *Cx3cl1*, and *Hsf1* (Figures 5E,F). Importantly, the number of dysregulated genes was reduced following nanosecond laser treatment, with only 3 genes showing reduced expression in the ApoE laser treated eye (*Mrpl43*, *Fbx16*, *Calb1*, Figures 5E,F). Similar to the changes in *Mertk* and *Pedf* (Figures 5B,C), this effect of laser treatment was also observed in the ApoEnull fellow eye, with only 6 genes showing reduced expression (Figures 5E,F). Grouping the dysregulated genes into major functional pathways, it can be seen that nanosecond laser treatment restores dysregulated genes associated with inflammatory response, telomere attrition, transcriptional regulation and proteostasis back to levels seen in the C57BL/6J aged RPE/choroid samples (Figure 5G).

**Figure 6:**
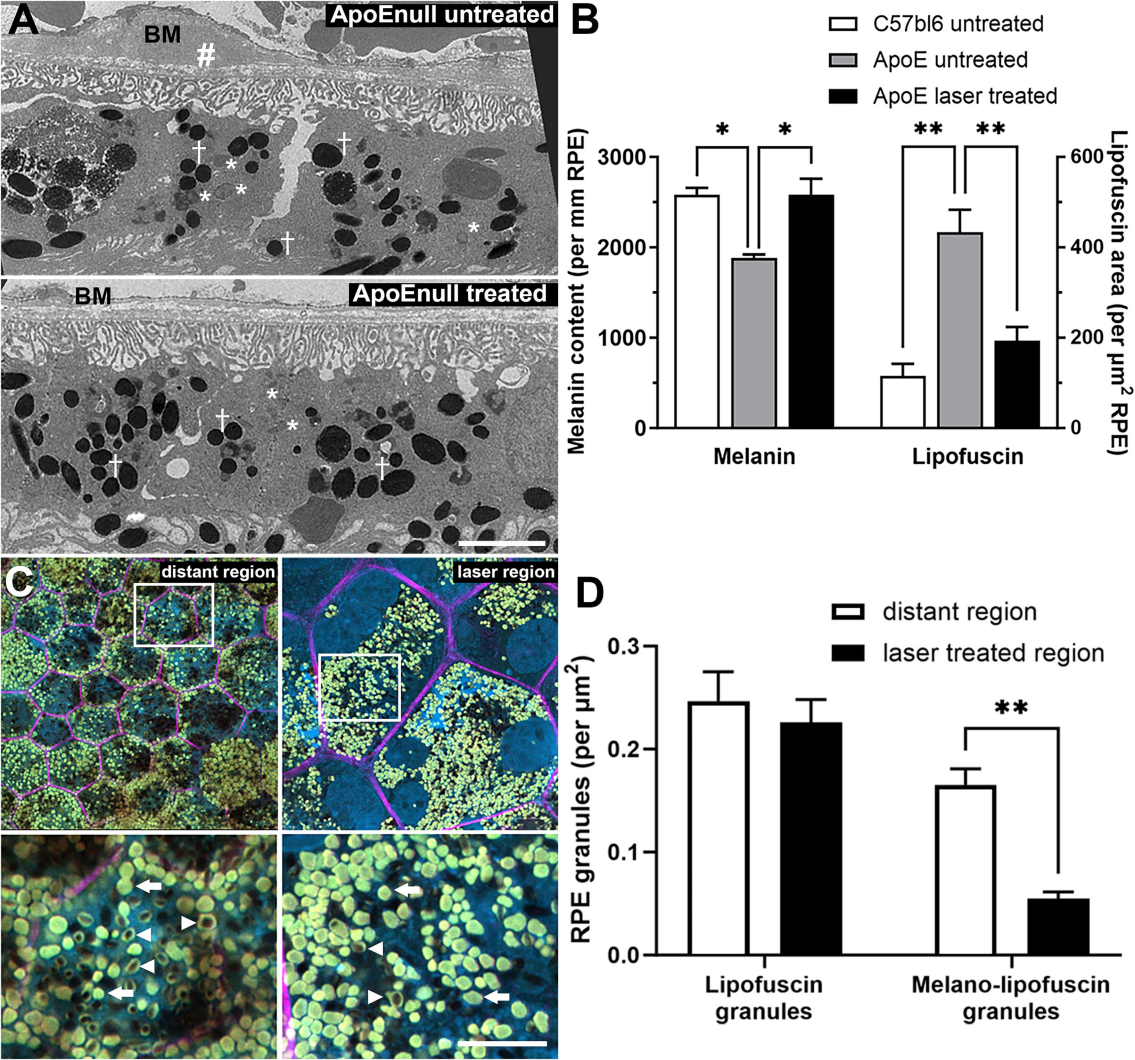
Reversal of RPE melanin and lipofuscin changes in aged ApoEnull mice and in human eyes treated with nanosecond laser. The capacity of the nanosecond laser to alter melanin and lipofuscin content was assessed in mice and human RPE. Aged C57BL/6J and ApoEnull mice were treated with nanosecond laser and after 3 months, melanin and lipofuscin content were quantified from TEM micrographs. **(A)** Representative TEM micrographs taken from ApoEnull untreated (top) and nanosecond laser treated (bottom) animals are shown with select melanin (crosses) and lipofuscin (asterisks) granules identified. The untreated ApoEnull image shows areas of thickened Bruch’s membrane (BM, #). **(B)** Grouped data were quantified and showed that melanin content was significantly reduced, while lipofuscin area was increased in the untreated ApoEnull RPE compared to age matched C57BL/6J RPE (one-way ANOVA, Tukey post hoc analysis). Nanosecond laser treatment increased melanin content and reduced lipofuscin area, with both measures restored to levels seen in age matched C57BL/6J controls (one-way ANOVA, Tukey post hoc analysis). **(C)** Representative human wholemount RPE images taken from laser treated (laser region) regions and those distant to the treated site (distant region) were stained with phalloidin-633 (pink) and bisbenzimide (blue). Areas of higher magnification taken from the upper panels (squares) are shown in the respective lower panels and show the presence of lipofuscin (arrow) and melanolipofuscin (arrowhead) granules. **(D)** Quantification of the RPE granules showed that nanosecond laser treatment did not alter the number of lipofuscin granules, however, did lead to a reduction in the number of melanolipofuscin content within laser-treated regions of the human RPE when compared to untreated (distant) RPE (2-way ANOVA, Šídák’s post hoc analsysis). Data shown as mean ± SEM, with *n* = 3 **(B)**, *n* ≥ 12 RPE cells **(D)**. *p < 0.05, **p < 0.01. BM, Bruch’s membrane, † melanin granules, * lipofuscin granules, # thickened Bruch’s membrane.

In order to determine whether these positive gene effects of the nanosecond laser were reflected in improved RPE cell health, C57BL/6J and ApoEnull RPE were imaged ultrastructurally to quantify melanin and lipofuscin content, both measures of RPE dysfunction and risk factors for AMD (35, 36). Representative RPE micrographs from control and laser treated ApoEnull animals are shown in Figure 6A and exhibit discrete intracellular vacuoles characteristic of melanin granules (†) and lipofuscin accumulation (*). The upper panel from the untreated ApoEnull cohort also exhibits an area of possible thickening of Bruch’s membrane (Figure 6A; BM, #) a feature that we have shown previously to occur in this model (11, 34). When melanin content was quantified, RPE in the ApoEnull untreated animals exhibited reduced levels, which were restored to C57BL/6J control levels upon laser treatment (Figure 6B; *p* < 0.01, one-way ANOVA). Similarly, the increased area of lipofuscin within the ApoEnull untreated RPE was reduced to age-matched C57BL/6J control levels after nanosecond laser treatment (Figure 6B; *p* < 0.01; one-way ANOVA).

Lipofuscin was also assessed in the human laser treated eye. RPE whole mounts were labeled with phalloidin (Figure 6C, pink) and the extent of lipofuscin (Figure 6C, arrows) and melanolipofuscin (Figure 6C, arrowheads) granules imaged at high resolution (bottom panels show magnified areas). When quantified (Figure 6D), there was no alteration in total lipofuscin granules between laser treated regions and those distant from the treatment site (*p* = 0.75, two way ANOVA), however, the number of melanolipofuscin granules was significantly reduced in laser treated regions (*p* < 0.01 for treated region, two-way ANOVA).

## 4 Discussion

The major findings of this study were that nanosecond laser treatment of mice was associated with RPE proliferation that was particularly pronounced within 3 days, although also observed up to 14 days post-treatment. RPE healing with possible proliferation was also observed in human treated tissue although over a longer time course (1–4 weeks). Moreover, a single nanosecond laser treatment was associated with sustained molecular changes in the RPE (3 months post treatment), with a significant enrichment of extracellular matrix gene sets and restoration of key RPE genes back to wild-type control levels in laser treated aged ApoEnull animals. Similarly, the number of dysregulated aging genes observed in the untreated ApoEnull samples was significantly reduced in laser treated and untreated fellow eyes. Finally, nanosecond laser treatment increased melanin content and reduced lipofuscin accumulation in the RPE of ApoEnull mice, while melanolipofuscin content was reduced within the treated human RPE cells. Overall, these data highlight that nanosecond laser treatment induces both short and longer term effects on the RPE that include abrogation of changes known to occur with age.

Our data show the RPE monolayer is rapidly healed in the mouse following nanosecond laser treatment, with significant proliferation (BrdU and Ki67 staining) aiding in the re-tiling of the monolayer within 3 days. The current data highlight a two-stage repair process governed by a rapid induction of proliferation to re-tile the monolayer (days 1–3), followed by a further limited induction of cell division to likely remodel the reformed region (day 14). While RPE are terminally differentiated, post-mitotic cells which rarely divide (37), this laser-induced proliferation of RPE is supported by our initial work highlighting cycinD1 staining following nanosecond laser (11) and that of others using *in vivo* and *in vitro* micropulse laser treatment (38–40). Interestingly, the data from our human eye which was treated with nanosecond laser at two time points, showed extended monolayer retiling characteristics of between 1 and 4 weeks.

While RPE structural integrity was restored rapidly post laser, the re-instatement of RPE barrier function was not assessed, despite the fact that F-actin (phalloidin) and tight junctions co-localize *in vitro* and *in vivo* at RPE cell junctions (41, 42). Previous work after nanosecond laser treatment has shown that RPE tight junctions (as assessed by zonula occludens-1) were restored between 3 and 7 days after *in vitro* and *in vivo* treatment (43). This laser-induced lesion / retiling is also accompanied by a limited inflammatory response, with previous work highlighting increases in inflammatory mediators (IL1β and TNFα) 6 h post-treatment, with expression returning to baseline by 24 h (44). Reflecting this limited inflammatory response, we have published that there is an increased interaction of microglial processes within the RPE lesion site, yet no gross change in microglial activation, nor expression of the complement protein, C3 (11).

One interesting comparison between the current data and that previously published, is the apparent increased repair rate following nanosecond laser treatment. While we show rapid RPE proliferation (1–3 days) and complete monolayer repair within 3 days, a previous *in vivo* study using SRT (micropulse) showed more delayed proliferation (peaking between day 3–7) and total repair by 7 days (38). *In vitro* based studies using micropulse laser treatment also show longer repair times to those reported here (27, 40). While it is difficult to compare across studies (e.g., total energy levels), the same mouse strain was used and both assessed repair within a 200 μm diameter treatment site. Therefore, it may be that the shorter laser duration of the nanosecond laser (3 ns compared to 1.7 μs in SRT) is able to promote increased RPE proliferation and a more rapid monolayer repair. Given the importance of an intact monolayer in maintaining the immune privilege of the sub retinal space and neural retina, shorter repair times may be advantageous (45). A direct *in vivo* comparison between subthreshold laser treatments would be required to confirm this.

One key outcome following nanosecond laser treatment, is that RPE cell proliferation was evident (although at a reduced level) in areas distant from the laser treatment site. Such distant effects on RPE cell proliferation after short pulse laser treatment are a novel finding. While similar distant effects in the treated eye have been reported following thermal laser photocoagulation, such proliferation likely arises due to the high thermal effect and accompanying collateral tissue damage (46). The fact that RPE proliferation occurred distant to the treatment site and after a longer time period post-laser (days 7 and 14) likely suggest the induction of an indirect signal rather than the direct laser effect. Previous work has indicated that numerous chemokines and growth factors are able to induce RPE proliferation such as fibroblast growth factor, epidermal growth factor and activation of the Wnt pathway (47–49). Quantification of RPE / retinal cytokine/growth factor profiles prior to day 7 may aid in identifying the mechanism underpinning this more generalized nanosecond laser effect on the treated eye.

In addition to the presence of RPE proliferation distant to the laser site in the treated eye, nanosecond laser treatment also induced RPE proliferation in the fellow, untreated eye. Previous work exploring the prophylactic use of lasers in AMD has reported reduced drusen load in the contralateral, untreated eye (11). Similarly, work in animal models of AMD (including the current study) show RPE gene expression changes in the fellow untreated eye that mirror those seen in the treated eye (11, 19). The RPE proliferation in the untreated eye may result in an improved RPE phenotype underpinning this fellow eye effect. This improved phenotype is supported by the current gene expression data and our previous work in aged ApoEnull animals (11). While a similar soluble cytokine/growth factor change as described above, could mediate this effect, a laser-induced change in systemic immune cells may also help drive this systemic change. Previous work using short pulse lasers has shown recruitment of retinal and systemic immune cells to the laser treated regions (11, 50), with evidence of altered cytokine expression reported in the RPE-choroid (50, 51). Based on such evidence, Caballero et al. suggest that the local and fellow eye response to short pulse laser may arise due to an immune cell mechanism (50).

The therapeutic potential of short pulse laser is thought to lie in the restoration of a “normal” RPE phenotype. This was explored in the current study by characterizing select gene changes and measures of cellular health after short- and longer-term periods post-treatment in normal wildtype mice (C57BL/6J) and in a model of AMD (ApoEnull mice). With respect to short (up to 7 days post-laser) and longer term (3 months post-laser) changes in otherwise normal mice, there was no obvious alteration in key RPE genes (*Rpe65*, *Mertk*, *Pedf*, and *Bdnf*), nor in the broader RPE transcriptome, although several pathways were overrepresented in the RNAseq dataset. Previous work following post-short pulse laser treatment have reported short term changes in RPE genes involved in angiogenesis (*Vegf*, *Pedf*), cytokine/inflammatory processes (*Tgfβ*, *Fgf2*), and matrix remodeling (Mmp2, Mmp9) (27, 39, 52, 53). To explain this apparent discrepancy, a couple of points should be highlighted. Firstly, most of the published work identifying RPE gene change have been undertaken using *in vitro* based tissues/cultured RPE cells rather than the *in vivo* design of the current work. While a couple of studies have explored gene expression in normal animals after *in vivo* treatment, control RPE gene expression was not presented in these studies due to the nature of the qPCR analysis undertaken (−2^ΔΔct−^, relative to wildtype controls) (40, 51). Secondly, most of the studies reporting a RPE gene expression change were undertaken prior to the complete restoration of the RPE monolayer (during the active re-tiling phase of the repair process), while gene expression in this study was assessed at 4 days after complete RPE retiling. Therefore, the identified gene changes in the previous studies may actually reflect the ongoing wound healing response, rather than any “improved” RPE phenotype.

While the current data showed no alteration in gene expression in normal animals, it did show alterations in the ApoEnull mouse. Specifically, we identify reduced *Mertk* and *Pedf* expression in untreated ApoEnull animals, with nanosecond laser treatment restoring expression to that seen in age-matched wildtype mice in both the treated and fellow untreated eyes. In addition, untreated ApoEnull animals exhibit dysregulation of numerous RPE-choroidal genes known to be involved in aging (24/84 genes), while laser treatment reduced the number of these dysregulated genes (3 genes in treated eye, 6 genes in fellow untreated eye). When these dysregulated genes were investigated in terms of biological process, a significant effect of the nanosecond laser was on age-related inflammatory genes (*Anxa5*, *C1s1*, *C5ar1*, *Cx3cl1*, *Fcer1g*, *Fcgr2b*, *Tmem135*), which were all restored to age-matched control levels after 3 months. While ApoE has been associated with an aging phenotype, particularly within the brain, these data also implicate a role for ApoE in RPE aging, which is associated with an increased risk for AMD (54). This is particularly relevant given particular APOE genotypes are associated with an increased risk for developing AMD (55, 56). Furthermore, to our knowledge, this is the first evidence that laser treatment can reverse this “aged” RPE phenotype. These data show nanosecond laser treatment alters the RPE transcriptome across numerous biological pathways and can be viewed in conjunction with previous work in the ApoEnull and Nrf2null models of AMD showing subthreshold laser to alter matrix remodeling (*Mmp2*, *Mmp3*) and inflammation (e.g., *FasL*, *IL1b*, *C3*) genes (11, 40, 51). Importantly, while this laser effect is widespread, it is by no means non-selective, with nanosecond laser only seeming to restore expression in those genes involved in the pathology found in the ApoEnull. Further work should look to correlate these gene related changes with altered protein expression after nanosecond laser treatment.

These laser-induced alterations in RPE proliferation and gene expression are also correlated with a change in RPE structure and pigment/waste processing. Particularly, we show nanosecond laser treatment restores melanin and lipofuscin content in ApoEnull RPE to age-matched wildtype levels. Melanin is a key light absorber within the RPE and reduced levels are indicative of ocular senescence and a risk factor for AMD, while accumulation of lipofuscin is observed in both aging and in AMD (35, 36). Importantly, we observe a similar effect in human RPE 1 month after nanosecond laser treatment, with the number of melanolipofuscin granules reduced in laser treated RPE cells compared to untreated neighboring regions. Previous work in humans have shown melanolipofuscin granules to predominate in normal and AMD foveal RPE cells, with recent evidence suggesting melanolipofuscin accumulates in AMD, while a reduction in lipofuscin is observed (26, 57). These findings suggest that nanosecond laser can restore melanin content and reduce the accumulation of phototoxic compounds, with the human data showing a rapid effect (≤1 month post treatment). When these data are combined with the evidence showing nanosecond and micropulse lasers can reduce Bruch’s membrane thickness, this type of treatment has the capacity to impact key biomarkers of AMD pathology (11, 16, 17).

While these data and those from our other studies highlight a potential therapeutic use for nanosecond laser in AMD (11, 14, 15, 19, 58), there are several factors that remain yet to be fully explored. For example, there is more work needed to determine the optimal number of treatment spots, laser dosage, and the interval between repeat interventions. To date, analysis of the LEAD data suggests no association between the number of nanosecond laser spots, nor laser energy on the efficacy of the laser (9). While it is unclear whether there is an optimal treatment window during disease progression, data do suggest that individual AMD phenotypes may impact on the efficacy of this treatment. For instance, those individuals with intermediate AMD and no reticular pseudodrusen showed a 4 fold decrease in progression to late AMD, while those with reticular pseudodrusen exhibited a trend for increased progression (15).

## 5 Conclusion

This work explored the response of murine and human RPE to nanosecond laser treatment. The data show nanosecond laser results in the formation of RPE with an improved phenotype, however, this only occurs in a pathological setting. The changes in the RPE transcriptome are relatively widespread and also result in the restoration of melanin content and a reduction in/redistribution of lipofuscin granules. As the RPE monolayer and its basement membrane undergo numerous structural and physiological changes during the progression of disease, this work provides a biological basis for the use of short pulse lasers as a treatment for AMD.

## Data Availability

The original contributions presented in the study are publicly available. These data have been deposited in NCBI’s Gene Expression Omnibus and are accessible through GEO Series accession number GSE287768 (https://www.ncbi.nlm.nih.gov/geo/query/acc.cgi?acc=GSE287768).

## References

[1] Guymer RH Campbell TG. Age-related macular degeneration. Lancet. (2023) 401:1459–72. doi: 10.1016/S0140-6736(22)02609-5 36996856

[2] Strauss O. The retinal pigment epithelium in visual function. Physiol Rev. (2005) 85:845–81. doi: 10.1152/physrev.00021.2004, PMID: 15987797

[3] Wimmers S Karl MO Strauss O. Ion channels in the RPE. Prog Retin Eye Res. (2007) 26:263–301. doi: 10.1016/j.preteyeres.2006.12.002, PMID: 17258931

[4] Wald G Brown PK. Synthesis and bleaching of rhodopsin. Nature. (1956) 177:174–6. doi: 10.1038/177174a0, PMID: 13297008

[5] Young RW Bok D. Participation of the retinal pigment epithelium in the rod outer segment renewal process. J Cell Biol. (1969) 42:392–403. doi: 10.1083/jcb.42.2.392, PMID: 5792328 PMC2107669

[6] Mitchell P Liew G Gopinath B Wong TY. Age-related macular degeneration. Lancet. (2018) 392:1147–59. doi: 10.1016/S0140-6736(18)31550-2, PMID: 30303083

[7] Wong JHC Ma JYW Jobling AI Brandli A Greferath U Fletcher EL. Exploring the pathogenesis of age-related macular degeneration: a review of the interplay between retinal pigment epithelium dysfunction and the innate immune system. Front Neurosci. (2022) 16:1009599. doi: 10.3389/fnins.2022.1009599, PMID: 36408381 PMC9670140

[8] Gass JD. Drusen and disciform macular detachment and degeneration. Arch Ophthalmol. (1973) 90:206–17. doi: 10.1001/archopht.1973.01000050208006, PMID: 4738143

[9] Cohn AC Wu Z Jobling AI Fletcher EL Guymer RH. Subthreshold Nano-second Laser treatment and age-related macular degeneration. J Clin Med. (2021) 10:484. doi: 10.3390/jcm10030484, PMID: 33525639 PMC7866172

[10] Wood JP Shibeeb O Plunkett M Casson RJ Chidlow G. Retinal damage profiles and neuronal effects of laser treatment: comparison of a conventional photocoagulator and a novel 3-nanosecond pulse laser. Invest Ophthalmol Vis Sci. (2013) 54:2305–18. doi: 10.1167/iovs.12-11203 23439601

[11] Jobling AI Guymer RH Vessey KA Greferath U Mills SA Brassington KH. Nanosecond laser therapy reverses pathologic and molecular changes in age-related macular degeneration without retinal damage. FASEB J. (2015) 29:696–710. doi: 10.1096/fj.14-262444, PMID: 25392267

[12] Framme C Schuele G Roider J Birngruber R Brinkmann R. Influence of pulse duration and pulse number in selective RPE laser treatment. Lasers Surg Med. (2004) 34:206–15. doi: 10.1002/lsm.20022, PMID: 15022247

[13] Neumann J Brinkmann R. Boiling nucleation on melanosomes and microbeads transiently heated by nanosecond and microsecond laser pulses. J Biomed Opt. (2005) 10:024001. doi: 10.1117/1.1896969, PMID: 15910075

[14] Lek JJ Brassington KH Luu CD Chen FK Arnold JJ Heriot WJ. Subthreshold nanosecond Laser intervention in intermediate age-related macular degeneration: study design and baseline characteristics of the Laser in early stages of age-related macular degeneration study (report number 1). Ophthalmol Retina. (2017) 1:227–39. doi: 10.1016/j.oret.2016.12.001, PMID: 31047426

[15] Guymer RH Wu Z Hodgson LAB Caruso E Brassington KH Tindill N. Intervention in early stages of age-related macular degeneration study, subthreshold nanosecond Laser intervention in age-related macular degeneration: the LEAD randomized controlled clinical trial. Ophthalmology. (2019) 126:829–38. doi: 10.1016/j.ophtha.2018.09.015, PMID: 30244144

[16] Tode J Richert E Koinzer S Klettner A von der Burchard C Brinkmann R. Selective retina therapy reduces Bruch's membrane thickness and retinal pigment epithelium pathology in age-related macular degeneration mouse models. Transl Vis Sci Technol. (2019) 8:11. doi: 10.1167/tvst.8.6.11, PMID: 31737435 PMC6855371

[17] Tode J Richert E Koinzer S Klettner A von der Burchard C Brinkmann R. Thermal stimulation of the retina reduces Bruch's membrane thickness in age related macular degeneration mouse models. Transl Vis Sci Technol. (2018) 7:2. doi: 10.1167/tvst.7.3.2, PMID: 29736323 PMC5931258

[18] Piedrahita JA Zhang SH Hagaman JR Oliver PM Maeda N. Generation of mice carrying a mutant apolipoprotein E gene inactivated by gene targeting in embryonic stem cells. Proc Natl Acad Sci USA. (1992) 89:4471–5. doi: 10.1073/pnas.89.10.4471, PMID: 1584779 PMC49104

[19] Guymer RH Brassington KH Dimitrov P Makeyeva G Plunkett M Xia W. Nanosecond-laser application in intermediate AMD: 12-month results of fundus appearance and macular function. Clin Experiment Ophthalmol. (2014) 42:466–79. doi: 10.1111/ceo.12247, PMID: 24118741

[20] Greferath U Guymer RH Vessey KA Brassington K Fletcher EL. Correlation of histologic features with *in vivo* imaging of reticular Pseudodrusen. Ophthalmology. (2016) 123:1320–31. doi: 10.1016/j.ophtha.2016.02.009, PMID: 27039021

[21] Jobling AI Vessey KA Waugh M Mills SA Fletcher EL. A naturally occurring mouse model of achromatopsia: characterization of the mutation in cone transducin and subsequent retinal phenotype. Invest Ophthalmol Vis Sci. (2013) 54:3350–9. doi: 10.1167/iovs.13-11831, PMID: 23580486

[22] Winer J Jung CK Shackel I Williams PM. Development and validation of real-time quantitative reverse transcriptase-polymerase chain reaction for monitoring gene expression in cardiac myocytes in vitro. Anal Biochem. (1999) 270:41–9. doi: 10.1006/abio.1999.4085, PMID: 10328763

[23] Reimand J Isserlin R Voisin V Kucera M Tannus-Lopes C Rostamianfar A. Pathway enrichment analysis and visualization of omics data using g: profiler, GSEA, Cytoscape and EnrichmentMap. Nat Protoc. (2019) 14:482–517. doi: 10.1038/s41596-018-0103-9, PMID: 30664679 PMC6607905

[24] Vessey KA Gu BJ Jobling AI Phipps JA Greferath U Tran MX. Loss of function of P2X7 receptor scavenger activity in aging mice: a novel model for investigating the early pathogenesis of age-related macular degeneration. Am J Pathol. (2017) 187:1670–85. doi: 10.1016/j.ajpath.2017.04.016, PMID: 28628761

[25] Julien S Biesemeier A Kokkinou D Eibl O Schraermeyer U. Zinc deficiency leads to lipofuscin accumulation in the retinal pigment epithelium of pigmented rats. PLoS One. (2011) 6:e29245. doi: 10.1371/journal.pone.0029245, PMID: 22216222 PMC3245262

[26] Bermond K Wobbe C Tarau IS Heintzmann R Hillenkamp J Curcio CA. Autofluorescent granules of the human retinal pigment epithelium: phenotypes, intracellular distribution, and age-related topography. Invest Ophthalmol Vis Sci. (2020) 61:35. doi: 10.1167/iovs.61.5.35, PMID: 32433758 PMC7405767

[27] Zhang JJ Sun Y Hussain AA Marshall J. Laser-mediated activation of human retinal pigment epithelial cells and concomitant release of matrix metalloproteinases. Invest Ophthalmol Vis Sci. (2012) 53:2928–37. doi: 10.1167/iovs.11-8585, PMID: 22447861

[28] Vessey KA Ho T Jobling AI Mills SA Tran MX Brandli A. Nanosecond Laser treatment for age-related macular degeneration does not induce focal vision loss or new vessel growth in the retina. Invest Ophthalmol Vis Sci. (2018) 59:731–45. doi: 10.1167/iovs.17-23098 29392319

[29] Bennis A Gorgels TG Ten Brink JB van der Spek PJ Bossers K Heine VM. Comparison of mouse and human retinal pigment epithelium gene expression profiles: potential implications for age-related macular degeneration. PLoS One. (2015) 10:e0141597. doi: 10.1371/journal.pone.0141597, PMID: 26517551 PMC4627757

[30] Booij JC van Soest S Swagemakers SM Essing AH Verkerk AJ van der Spek PJ. Functional annotation of the human retinal pigment epithelium transcriptome. BMC Genomics. (2009) 10:164. doi: 10.1186/1471-2164-10-164, PMID: 19379482 PMC2679759

[31] King R Lu L Williams RW Geisert EE. Transcriptome networks in the mouse retina: an exon level BXD RI database. Mol Vis. (2015) 21:1235–51. PMID: 26604663 PMC4626778

[32] Zhou RM Wang XQ Yao J Shen Y Chen SN Yang H. Identification and characterization of proliferative retinopathy-related long noncoding RNAs. Biochem Biophys Res Commun. (2015) 465:324–30. doi: 10.1016/j.bbrc.2015.07.120, PMID: 26241674

[33] Subramanian A Tamayo P Mootha VK Mukherjee S Ebert BL Gillette MA. Gene set enrichment analysis: a knowledge-based approach for interpreting genome-wide expression profiles. Proc Natl Acad Sci USA. (2005) 102:15545–50. doi: 10.1073/pnas.0506580102, PMID: 16199517 PMC1239896

[34] Vessey KA Jobling AI Tran MX Wang AY Greferath U Fletcher EL. Treatments targeting autophagy ameliorate the age-related macular degeneration phenotype in mice lacking APOE (apolipoprotein E). Autophagy. (2022) 18:2368–84. doi: 10.1080/15548627.2022.2034131, PMID: 35196199 PMC9542759

[35] Sarna T Burke JM Korytowski W Rozanowska M Skumatz CM Zareba A. Loss of melanin from human RPE with aging: possible role of melanin photooxidation. Exp Eye Res. (2003) 76:89–98. doi: 10.1016/S0014-4835(02)00247-6, PMID: 12589778

[36] Katz ML. Potential role of retinal pigment epithelial lipofuscin accumulation in age-related macular degeneration. Arch Gerontol Geriatr. (2002) 34:359–70. doi: 10.1016/S0167-4943(02)00012-2, PMID: 14764336

[37] Blenkinsop TA Saini JS Maminishkis A Bharti K Wan Q Banzon T. Human adult retinal pigment epithelial stem cell-derived RPE monolayers exhibit key physiological characteristics of native tissue. Invest Ophthalmol Vis Sci. (2015) 56:7085–99. doi: 10.1167/iovs.14-16246, PMID: 26540654 PMC4640474

[38] Kim HD Jang SY Lee SH Kim YS Ohn YH Brinkmann R. Retinal pigment epithelium responses to selective retina therapy in mouse eyes. Invest Ophthalmol Vis Sci. (2016) 57:3486–95. doi: 10.1167/iovs.16-19508, PMID: 27367516

[39] Richert E Koinzer S Tode J Schlott K Brinkmann R Hillenkamp J. Release of different cell mediators during retinal pigment epithelium regeneration following selective retina therapy. Invest Ophthalmol Vis Sci. (2018) 59:1323–31. doi: 10.1167/iovs.17-23163, PMID: 29625455

[40] Richert E Papenkort J von der Burchard C Klettner A Arnold P Lucius R. Selective retina therapy and thermal stimulation of the retina: different regenerative properties - implications for AMD therapy. BMC Ophthalmol. (2021) 21:412. doi: 10.1186/s12886-021-02188-8, PMID: 34847865 PMC8630886

[41] Ban Y Rizzolo LJ. A culture model of development reveals multiple properties of RPE tight junctions. Mol Vis. (1997) 3:18. PMID: 9479009

[42] Narimatsu T Ozawa Y Miyake S Kubota S Hirasawa M Nagai N. Disruption of cell-cell junctions and induction of pathological cytokines in the retinal pigment epithelium of light-exposed mice. Invest Ophthalmol Vis Sci. (2013) 54:4555–62. doi: 10.1167/iovs.12-11572, PMID: 23761083

[43] Wood JPM Tahmasebi M Casson RJ Plunkett M Chidlow G. Physiological response of the retinal pigmented epithelium to 3-ns pulse laser application, *in vitro* and *in vivo*. Clin Experiment Ophthalmol. (2021) 49:454–69. doi: 10.1111/ceo.13931 33904222

[44] Chidlow G Shibeeb O Plunkett M Casson RJ Wood JP. Glial cell and inflammatory responses to retinal laser treatment: comparison of a conventional photocoagulator and a novel, 3-nanosecond pulse laser. Invest Ophthalmol Vis Sci. (2013) 54:2319–32. doi: 10.1167/iovs.12-11204 23439603

[45] Du Y Yan B. Ocular immune privilege and retinal pigment epithelial cells. J Leukoc Biol. (2023) 113:288–304. doi: 10.1093/jleuko/qiac016, PMID: 36805720

[46] von Leithner PL Ciurtin C Jeffery G. Microscopic mammalian retinal pigment epithelium lesions induce widespread proliferation with differences in magnitude between center and periphery. Mol Vis. (2010) 16:570–81. 20360994 PMC2847682

[47] Steindl-Kuscher K Boulton ME Haas P Dossenbach-Glaninger A Feichtinger H Binder S. Epidermal growth factor: the driving force in initiation of RPE cell proliferation. Graefes Arch Clin Exp Ophthalmol. (2011) 249:1195–200. doi: 10.1007/s00417-011-1673-1, PMID: 21494877 PMC3714846

[48] Hanovice NJ Leach LL Slater K Gabriel AE Romanovicz D Shao E. Regeneration of the zebrafish retinal pigment epithelium after widespread genetic ablation. PLoS Genet. (2019) 15:e1007939. doi: 10.1371/journal.pgen.1007939, PMID: 30695061 PMC6368336

[49] Amann J Kaven C Spraul CW Lang GK Lang GE. Effect of octreotide combined with growth factors on proliferation of RPE cells in vitro. Ophthalmologe. (2000) 97:737–41. doi: 10.1007/s003470070020, PMID: 11130160

[50] Caballero S Kent DL Sengupta N Li Calzi S Shaw L Beli E. Bone marrow-derived cell recruitment to the neurosensory retina and retinal pigment epithelial cell layer following subthreshold retinal phototherapy. Invest Ophthalmol Vis Sci. (2017) 58:5164–76. doi: 10.1167/iovs.16-20736, PMID: 29049716 PMC5636205

[51] Richert E von der Burchard C Klettner A Arnold P Lucius R Brinkmann R. Modulation of inflammatory processes by thermal stimulating and RPE regenerative laser therapies in age related macular degeneration mouse models. Cytokine X. (2020) 2:100031. doi: 10.1016/j.cytox.2020.100031, PMID: 33604557 PMC7885883

[52] Li Z Song Y Chen X Chen Z Ding Q. Biological modulation of mouse RPE cells in response to subthreshold diode micropulse Laser treatment. Cell Biochem Biophys. (2015) 73:545–52. doi: 10.1007/s12013-015-0675-8, PMID: 27352351

[53] Dang Y Wu W Xu Y Mu Y Xu K Wu H. Effects of low-level laser irradiation on proliferation and functional protein expression in human RPE cells. Lasers Med Sci. (2015) 30:2295–302. doi: 10.1007/s10103-015-1809-3, PMID: 26404781

[54] Yassine HN Finch CE. APOE Alleles and Diet in Brain Aging and Alzheimer's Disease. Front Aging Neurosci. (2020) 12:150. doi: 10.3389/fnagi.2020.00150, PMID: 32587511 PMC7297981

[55] Fritsche LG Freitag-Wolf S Bettecken T Meitinger T Keilhauer CN Krawczak M. Age-related macular degeneration and functional promoter and coding variants of the apolipoprotein E gene. Hum Mutat. (2009) 30:1048–53. doi: 10.1002/humu.20957, PMID: 19384966

[56] Baird PN Richardson AJ Robman LD Dimitrov PN Tikellis G McCarty CA. Apolipoprotein (APOE) gene is associated with progression of age-related macular degeneration (AMD). Hum Mutat. (2006) 27:337–42. doi: 10.1002/humu.20288, PMID: 16453339

[57] Bermond K von der Emde L Tarau IS Bourauel L Heintzmann R Holz FG. Autofluorescent organelles within the retinal pigment epithelium in human donor eyes with and without age-related macular degeneration. Invest Ophthalmol Vis Sci. (2022) 63:23. doi: 10.1167/iovs.63.1.23, PMID: 35050307 PMC8787573

[58] Wu Z Luu CD Hodgson LAB Caruso E Brassington KH Tindill N. Secondary and exploratory outcomes of the subthreshold nanosecond laser intervention randomized trial in age-related macular degeneration: a LEAD study report. Ophthalmol Retina. (2019) 3:1026–34. doi: 10.1016/j.oret.2019.07.008, PMID: 31582304

